# Multi-classification model incorporating radiomics and clinic-radiological features for predicting invasiveness and differentiation of pulmonary adenocarcinoma nodules

**DOI:** 10.1186/s12938-023-01180-1

**Published:** 2023-11-30

**Authors:** Haitao Sun, Chunling Zhang, Aimei Ouyang, Zhengjun Dai, Peiji Song, Jian Yao

**Affiliations:** 1 Medical Imaging Center, Central Hospital Affiliated to Shandong First Medical University, 105 Jiefang Road, Lixia District, Jinan, 250013 Shandong Province China; 2 Scientific Research Department of Huiying Medical Technology Co., Ltd, 66 Xixiaokou Road, Haidian District, Beijing, 100192 China; 3 Medical Imaging Center, Shandong Provincial Hospital Affiliated to Shandong First Medical University, 324 Jingwuweiqi Road, Huaiyin District, Jinan, 250021 Shandong Province China

**Keywords:** Computed tomography, Radiomics, Machine learning, Pulmonary nodule, Multi-classification

## Abstract

**Purpose:**

To develop a comprehensive multi-classification model that combines radiomics and clinic-radiological features to accurately predict the invasiveness and differentiation of pulmonary adenocarcinoma nodules.

**Methods:**

A retrospective analysis was conducted on a cohort comprising 500 patients diagnosed with lung adenocarcinoma between January 2020 and December 2022. The dataset included preoperative CT images and histological reports of adenocarcinoma in situ (AIS, *n* = 97), minimally invasive adenocarcinoma (MIA, *n* = 139), and invasive adenocarcinoma (IAC, *n* = 264) with well-differentiated (WIAC, *n* = 99), moderately differentiated (MIAC, *n* = 84), and poorly differentiated IAC (PIAC, *n* = 81). The patients were classified into two groups (IAC and non-IAC) for binary classification and further divided into three and five groups for multi-classification. Feature selection was performed using the least absolute shrinkage and selection operator (LASSO) algorithm to identify the most informative radiomics and clinic-radiological features. Eight machine learning (ML) models were developed using these features, and their performance was evaluated using accuracy (ACC) and the area under the receiver-operating characteristic curve (AUC).

**Results:**

The combined model, utilizing the support vector machine (SVM) algorithm, demonstrated improved performance in the testing cohort, achieving an AUC of 0.942 and an ACC of 0.894 for the two-classification task. For the three- and five-classification tasks, the combined model employing the one versus one strategy of SVM (SVM-OVO) outperformed other models, with ACC values of 0.767 and 0.607, respectively. The AUC values for histological subtypes ranged from 0.787 to 0.929 in the testing cohort, while the Macro-AUC and Micro-AUC of the multi-classification models ranged from 0.858 to 0.896.

**Conclusions:**

A multi-classification radiomics model combined with clinic-radiological features, using the SVM-OVO algorithm, holds promise for accurately predicting the histological characteristics of pulmonary adenocarcinoma nodules, which contributes to personalized treatment strategies for patients with lung adenocarcinoma.

## Introduction

Pulmonary nodules are prevalent during CT screening, with at least one nodule detected in up to 51% of initial screenings [1]. Despite over 95% of the nodules being ultimately determined as benign, a significant number of malignant pulmonary nodules are still detected due to the vast number of cases screened [2, 3]. Lung adenocarcinoma (LAC) is the dominant histological subtype of malignant pulmonary nodules [4, 5]. As per the pathological classification by the World Health Organization, LAC is categorized into three types based on the level of invasiveness—adenocarcinoma in situ (AIS), minimally invasive adenocarcinoma (MIA), and invasive adenocarcinoma (IAC) [6]. In 2020, the International Association for the Study of Lung Cancer (IASLC) reclassified IAC into three grades based on varying levels of differentiation including well-differentiated IAC (WIAC), moderately differentiated IAC (MIAC), and poorly differentiated IAC (PIAC), which proved superior to models incorporating nuclear or cytologic grade [7]. Each LAC subtype exhibits distinct biological characteristics and prognosis. As a preinvasive lesion, AIS is typically managed through follow-up surveillance, but some nodules may progress to MIA or IAC [8]. AIS and MIA perform the excellent prognosis after sub-lobar resection [9], while poorly and moderately differentiated IAC exhibit higher postoperative recurrence rates compared to well-differentiated IAC [7, 10]. Therefore, accurately identifying the invasiveness and differentiation of adenocarcinoma to classify LAC may provide guidance for surveillance, surgical strategy, and prognosis based on preoperative CT images.

Traditionally, the classification of LAC subtypes relies on visual assessment and verbal description of radiological features. Previous investigations have highlighted the significance of nodule characteristics, including size, type, margin, pleural indentation, vacuole sign, and vascular convergence sign, in determining the pathological nature of pulmonary nodules [11–14]. However, accurate classification heavily relies on the expertise and diagnostic proficiency of radiologists. Li et al. found that senior radiologists exhibited superior predictive capabilities in discerning the grading of IAC compared to their junior counterparts [15]. Presently, histological classification necessitates invasive tissue sampling through surgery or needle biopsy, which can be burdensome. Therefore, the development of a non-invasive and convenient approach to anticipate the histological subtypes of pulmonary nodules holds significant clinical implications.

Radiomics, a highly promising methodology, involves the extraction of numerous high-dimensional, retrievable features from medical imaging data, either independently or in conjunction with clinical features [16–18]. This approach has shown utility in distinguishing between benign and malignant lung nodules, predicting the invasiveness of lung adenocarcinoma, and identifying the preoperative IASLC grade of IAC [19–22]. Several studies have developed radiomics models to categorize and predict the pathological attributes of specific nodules, such as pure ground-glass nodules (pGGNs), mixed ground-glass nodules (mGGNs), and solid nodules (SNs), demonstrating robust predictive capabilities [23–25]. However, these studies have primarily focused on two or three-classification radiomics, thus lacking coverage of the majority of nodule types and new pathological gradings. Therefore, the development of a multi-classification radiomics approach that can predict the pathological invasiveness and differentiation of pulmonary nodules holds greater clinical value and practicality [26].

With this foundation in mind, the objective of this investigation is to construct a five-classification radiomics model integrating clinic-radiological features for the prediction of invasiveness and differentiation of adenocarcinoma nodules, encompassing AIS, MIA, WIAC, MIAC, and PIAC. The ultimate aim is to establish a non-invasive approach that enables comprehensive assessment of the histological classifications of pulmonary nodules.

## Materials and methods

### Patients

**Fig. 1:**
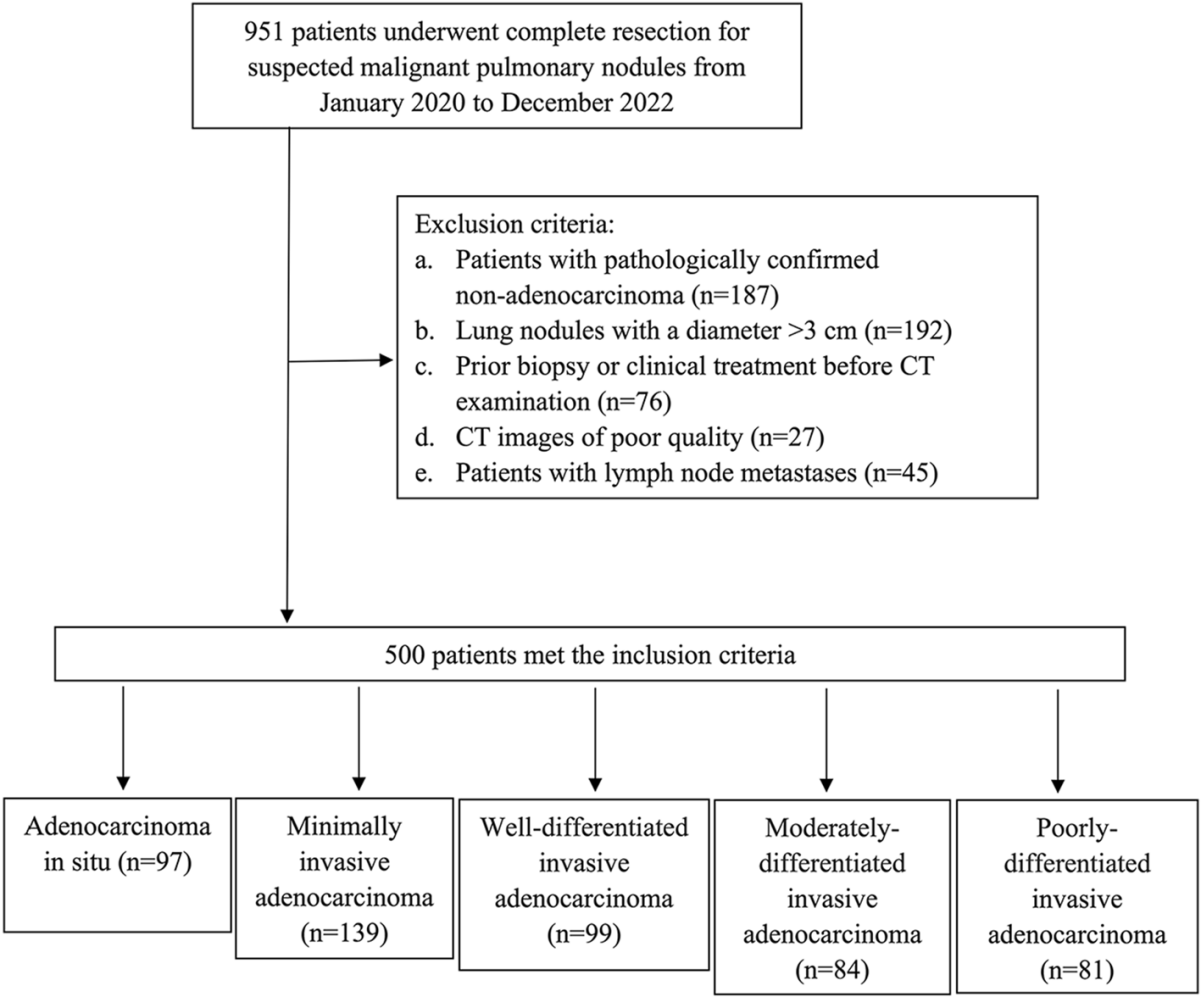
Flowchart of the patient selection

A total of 951 patients who underwent complete resection for suspected lung cancer were included in this study, with clinical data and preoperative CT images collected between January 2020 and December 2022. To enhance the homogeneity of the patient cohorts, specific exclusion criteria were applied, which encompassed: (1) patients with confirmed non-adenocarcinoma histology, such as squamous carcinoma, mucinous adenocarcinoma, metastases, and others (*n* = 187); (2) lung nodules larger than 3 cm in diameter (*n* = 192); (3) patients who received clinical treatment and needle biopsy prior to the CT examination (*n* = 76); (4) CT images of inadequate quality (*n* = 27); and (5) patients with lymph-node metastases (*n* = 45). Ultimately, a total of 500 patients (201 men and 299 women) were retrospectively enrolled in this study, with a median age of 59 years (age range: 19–83 years). Among these patients, there were 97 cases of AIS, 139 cases of MIA, 99 cases of WIAC, 84 cases of MIAC, and 81 cases of PIAC. The detailed process of patient recruitment is presented in Fig. 1.

### Histopathological evaluation

All lung specimens that underwent surgical resection were meticulously examined following the 2021 WHO classification of thoracic tumors and the newly proposed grading system by the IASLC [6, 7]. The diagnosis of LAC was made based on comprehensive histologic patterns, which encompassed lepidic, acinar, papillary, micropapillary, solid, cribriform, and complex glandular patterns [27]. The proportion of each histologic pattern was recorded in 5% increments to determine the predominant histologic subtype and quantify any patterns for tumor grading. Adenocarcinoma was categorized into two groups based on the degree of invasion: non-IAC (including AIS and MIA) and IAC (including WIAC, MIAC, and PIAC). Within the IAC group, low-grade subtypes were further divided into WIAC and MIAC, while high-grade subtypes (PIAC) were also considered. This grading scheme was showed in Table 1 [7]. According to the pathological invasiveness and differentiation of pulmonary nodules, the definition of two-, three-, five-classification task was as follows: two-classification was (AIS, MIA) vs (WIAC, MIAC, and PIAC), three-classification was (AIS, MIA) vs (WIAC, MIAC) vs (PIAC), and five-classification was (AIS) vs (MIA) vs (WIAC) vs (MIAC) vs (PIAC).

**Table 1:** Grading scheme for invasive adenocarcinomas of pulmonary nodules

Grade	Differentiation	Patterns
1	Well differentiated	Lepidic predominant with no or less than 20% of high-grade patterns
2	Moderately differentiated	Acinar or papillary predominant with no or less than 20% of high-grade patterns
3	Poorly differentiated	Any tumor with 20% or more of high-grade patterns

High-grade patterns include solid, micropapillary, and complex glandular patterns

### CT acquisition

The patients underwent CT plain imaging of the lungs using one of three CT systems: Somatom Definition AS 64 (Siemens Healthcare, Germany), Somatom Definition Flash (Siemens Healthcare, Germany), GE Discovery CT750 HD (GE Medical Systems, USA). Patients were scanned in the supine position with complete inspiration and breath-holding, from the apex of the lung to the diaphragm. The scanning process utilized a tube voltage of 120 kV, automatic tube current, and reconstruction slice thickness and interval of 1 or 1.25 mm. Reconstruction settings included a lung window with a mean of -500 HU and a width of 1500 HU, with a matrix size of 512 × 512.

### Clinical and radiological features

The clinical data, including gender, age, smoking history, hypertension, diabetes, and neoplasia history, was retrieved from the hospital information system. The radiological characteristics were assessed by two thoracic specialists, and a consensus was reached. These characteristics encompassed the involved lobe (right upper lobe, right middle lobe, right lower lobe, left upper lobe, and left lower lobe), nodule shape (regular and irregular), nodule type (including pGGNs, mGGNs, and SNs), boundary (clear or blurred border definition), lobulation (indentation at the edge of a round or oval lesion), speculation (linear strands extending into the lung parenchyma but not touching the pleural surface), vacuole (small focal areas of low attenuation within the nodule), air bronchogram (tubelike or branched air structure within the nodule), vascular convergence (multiple supplying vessels converging toward the lesion), pleural retraction (linear strands extending toward the pleura or major/minor fissure from the mass, causing pleural distortion), bronchial cut-off (sudden truncation of a bronchus due to obstruction within the nodule), and presence of abnormal vessels within the nodules (distorted, dilated, and complicated vessels within the lesions) [14, 28–30].

### Pulmonary nodules’ segmentation

A single radiologist (with 10 years of experience in chest imaging), who was blinded to the pathological results, conducted semi-automated lesion segmentation on CT images with lung window settings using the Radcloud Platform [31] (version 7.5; Huiying Medical Technology Co., Ltd., Beijing). The segmentation process involved delineating the pulmonary nodules on a section-by-section basis to generate a three-dimensional region of interest (ROI). After a month, 50 cases were randomly selected, and the same radiologist repeated the segmentation to assess intra- and interobserver reproducibility. The aforementioned segmentation results were further validated by an experienced radiologist (with 15 years of experience in chest imaging).

### Radiomics and clinic-radiological features extraction and selection

The extraction of image features plays a fundamental and crucial role in radiomics analysis, as it enables the identification of relevant features that effectively capture the biological characteristics of lesions and tumor heterogeneity. In this study, the Radcloud platform was utilized for the extraction of radiomics features. Specifically, the platform employed PyRadiomics [32] (version 3.1.0, https://pyradiomics.readthedocs.io/), a Python-based library, to extract a comprehensive set of radiomics features from the medical images. To enhance the reproducibility of the radiomics analyses, pre-processing steps were meticulously addressed. Prior to feature extraction, $z$ normalization of CT images was conducted using PyRadiomics. Additionally, grayscale discretization employed fixed Bin Width values set at 25HU, and voxel size resampling was executed at 1 × 1 × 1 mm^3^ using PyRadiomics [33].

In our study, we extracted a total of 1688 image features belonging to five major categories from the ROI of each patient. These categories include first-order statistics, 3D shape features, gray-level co-occurrence matrix (GLCM) features, gray-level run length matrix (GLRL) features, gray-level size zone matrix (GLSZM) features, neighboring gray tone difference matrix (NGTDM) features, and gray-level dependence matrix (GLDM) features. Notably, shape features were solely derived from the original images, while the remaining features were obtained by applying various filters such as wavelet, square, square root, gradient, logarithm, exponential, local binary pattern in 2D (LBP-2D), and local binary pattern in 3D (LBP-3D). For the extraction of texture features, preprocessed CT images underwent wavelet filtering, which involved transforming the VOI into the wavelet domain while preserving low-pass (LLL) and high-pass (HHH) subbands and assigning different weights to other subbands (LHL, LHH, LLH, HLL, HHL, and HLH). Additionally, the LBP-3D image type comprised three subcategories, including the kurtosis map (LBP-3D-k), as well as two categories calculated using different levels of spherical harmonics, namely LBP-3D-m1 and LBP-3D-m2. It is worth mentioning that all the aforementioned radiological features adhere to the Image Biomarker Standardization Initiative (IBSI, https://theibsi.github.io).

Prior to selecting radiomics features, *Z*-score normalization was applied to all features. Each patient possessed a total of 1688 features, resulting in a significant amount of redundancy. To avoid diminishing the predictive performance of the model and to reduce computational time, it is necessary to perform feature selection in training set before model development. First, the evaluation of interobserver reproducibility for radiomics features was conducted utilizing the intraclass correlation coefficient (ICC). Specifically, ICC values falling below 0.5, between 0.5 and 0.75, between 0.75 and 0.9, and exceeding 0.90 are indicative of poor, moderate, good, and excellent reliability, respectively [34]. Consequently, features with ICC values surpassing 0.75 were retained for subsequent stages of feature selection. Subsequently, a variance threshold of 0.8 was employed to refine the feature selection process. Furthermore, the univariate analysis method, SelectKBest, was utilized to identify features with a *p* value less than 0.05 for further analysis. Finally, the least absolute shrinkage and selection operator (LASSO) regression method with tenfold cross-validation was employed to assist in feature selection, aiming to identify relevant and informative features associated with lung cancer classification.

On the other hand, LASSO regression is also applicable for the selection of clinic-radiological features [35], integrating radiological scores with independent clinical risk factor scores to establish a predictive model for lung cancer classification. Specifically, LASSO shrinks all regression coefficients close to zero based on a regularization parameter *λ* and precisely sets the coefficients of many irrelevant features to zero. To determine the optimal value of *λ*, we employed a tenfold cross-validation with a minimum criterion, resulting in the *λ* value that yielded the lowest cross-validation error. The retained non-zero coefficient features were used to fit the regression model and combined into a radiomics and clinic-radiological features model. The predicted values of the model for each patient were computed through a linear combination weighted by the correlation coefficients of the selected features.

Given the involvement of two-classification, three-classification, and five-classification tasks in our study, it is essential to note that the LASSO labels employed during feature selection are tailored to the specific categorization requirements of each respective task. In other words, the labels used in the LASSO regularization process correspond uniquely to the distinct classification schemes associated with the two-classification, three-classification, and five-classification tasks. This tailored approach ensures the relevance and appropriateness of the selected features for each specific classification task within our study framework.

### Development of machine learning (ML) models

The features extracted from lesion segmentation using computer learning techniques are subjected to data analysis and model construction, enabling the reflection of lesion information and prediction of the lesions. The radiomics dataset comprises a training set for training and a testing set for model testing. Currently, commonly used radiomics models include logistic regression (LR), support vector machine (SVM), K-nearest neighbors (KNN), decision tree (DT), random forest (RF), gradient boosting decision tree (GBDT), among others. One versus rest (OVR) and one versus one (OVO) are two well-known strategies that decompose multi-class classification problems into multiple binary classification problems. Since LR and SVM are two-classification models, this study categorizes them into LR-OVR, SVM-OVR, LR-OVO, and SVM-OVO for three-classification and five-classification tasks, while also comparing them with other multi-classification models, such as KNN, DT, RF, and GBDT.

### Validation of the optimizing ML models

**Fig. 2:**
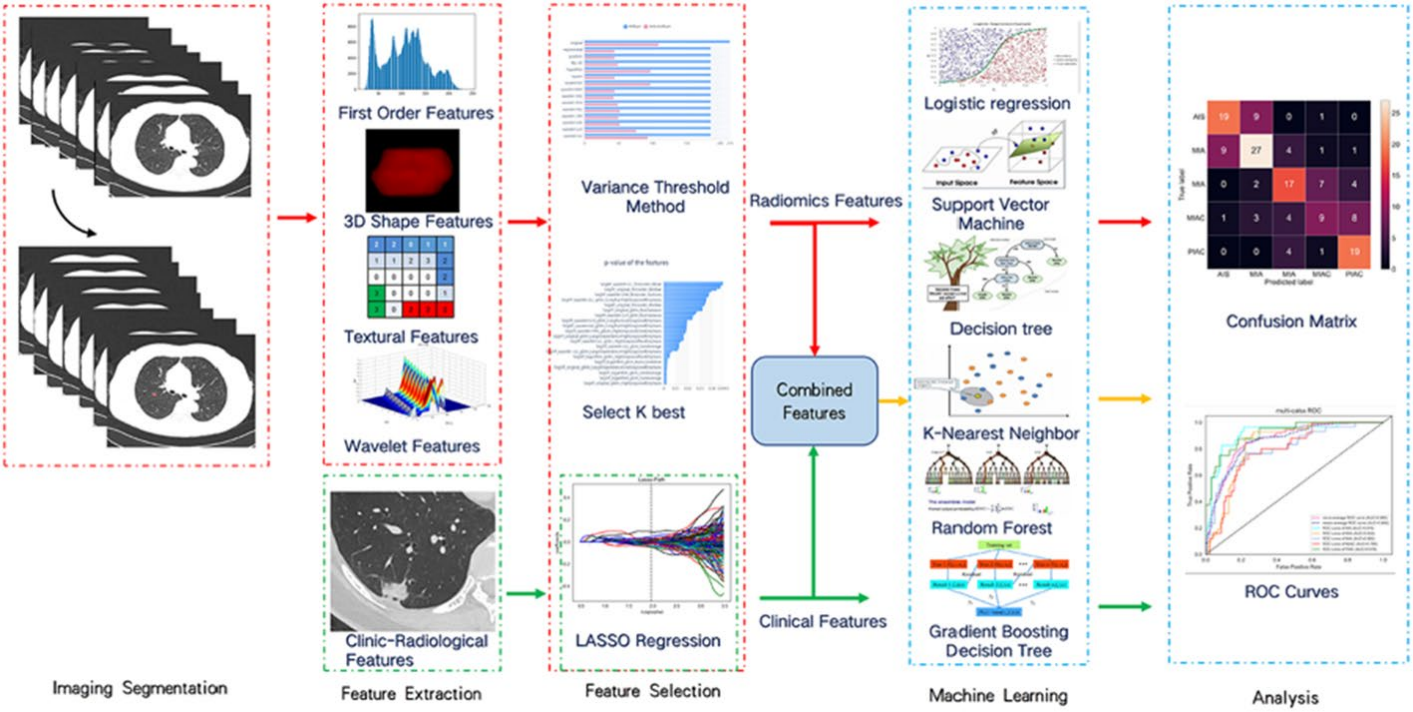
Workflow of necessary steps in current study. LASSO least absolute shrinkage and selection operator, *ROC* receiver-operating characteristic

In the evaluation of the testing cohort, the performance of the two-classification problem was assessed using quantitative measures, such as accuracy (ACC), sensitivity, specificity, and the area under the receiver-operating characteristic curve (AUC). Furthermore, considering the inherent characteristics of the multi-classification problem, the predictive capabilities of the models designed for multiple classes were examined by computing macro- and micro-averaged AUCs [36]. Additionally, macro-average accuracy, F1-score, recall, and precision were calculated to evaluate the classification performance of the multi-classification models. Notably, in addition to the radiomics model, the same methodology was employed to develop the clinic-radiological model and combined model, with the objective of verifying whether the inclusion of clinical variables enhances the classification performance of the machine learning models for pulmonary nodules. Our study flow diagram is shown in Fig. 2.

### Statistical analyses

The statistical analyses were conducted using R software (version 4.2.1; https://www.r-project.org/) to compare the differences in clinical and radiological data among the five groups. For categorical variables, the Chi-square test was employed, and in terms of quantitative variables, the Mann–Whitney *U* test was applied. The overall performance of the multi-classification models in the development and testing cohorts was evaluated through receiver-operating characteristic (ROC) curve analysis and the calculation of micro- and macro-AUC. All statistical tests were two-sided, and a significance level of P < 0.05 was deemed statistically significant throughout the entire study duration.

## Results

### Patient characteristics

Table 2 displays the comprehensive clinical and radiological features observed in a cohort of 500 patients. The enrolled patients underwent random allocation into a training set (*n* = 349) and a testing set (*n* = 151) at a ratio of 7:3. Statistical analysis revealed significant differences among the five histological subtypes for various clinical and radiological features, including gender, smoking history, hypertension, age, nodule boundary, lobulation sign, bronchial cut-off sign, speculation sign, vacuole sign, air bronchogram sign, vascular convergence sign, pleural retraction, abnormal vessels within nodules, and nodule type (*p* < 0.05).

**Table 2:** The clinical and radiological features of patients in the histological classifications

Variable		Total (*n* = 500)	AIS (*n* = 97)	MIA (*n* = 139)	WIAC (*n* = 99)	MIAC (*n* = 84)	PIAC (*n* = 81)	*p*
Gender, *n* (%)	Male	201 (40)	31 (32)	46 (33)	38 (38)	33 (39)	51 (63)	< 0.001^*^
	female	299 (60)	66 (68)	91 (65)	61 (62)	51 (61)	30 (37)	
Smoking history, *n* (%)	No	384 (77)	86 (89)	116 (83)	76 (77)	62 (74)	44 (54)	< 0.001^*^
	Yes	116 (23)	11 (11)	23 (17)	23 (23)	22 (26)	37 (46)	
Hypertension, *n* (%)	No	308 (62)	69 (71)	93 (67)	54 (55)	48 (57)	44 (54)	0.041^*^
	Yes	192 (38)	28 (29)	46 (33)	45 (45)	36 (43)	37 (46)	
Diabetes, *n* (%)	No	427 (85)	85 (88)	119 (86)	84 (85)	71 (85)	68 (84)	0.963
	Yes	73 (15)	12 (12)	20 (14)	15 (15)	13 (15)	13 (16)	
History of tumor, *n* (%)	No	462 (92)	88 (91)	127 (91)	97 (98)	76 (90)	74 (91)	0.235
	Yes	38 (8)	9 (9)	12 (9)	2 (2)	8 (10)	7 (9)	
Nodule involved lobe, *n* (%)	RUL	187 (37)	40 (41)	42 (30)	45 (45)	28 (33)	32 (40)	0.218
	RML	31 (6)	8 (8)	7 (5)	3 (3)	7 (8)	6 (7)	
	RLL	93 (19)	22 (23)	24 (17)	18 (18)	14 (17)	15 (19)	
	LUL	108 (22)	12 (12)	41 (29)	21 (21)	17 (20)	17 (21)	
	LLL	81 (16)	15 (15)	25 (18)	12 (12)	18 (21)	11 (14)	
Age, median (Q1,Q3)		59(52, 67)	55(48, 59)	57(50, 64)	64(58, 70)	62(55, 68)	65(59, 70)	< 0.001^§^
Boundary, *n* (%)	Ill-defined	109 (22)	36 (37)	29 (21)	21 (21)	13 (15)	10 (12)	< 0.001^*^
	Well-defined	391 (78)	61 (63)	110 (79)	78 (79)	71 (85)	71 (88)	
Lobulation sign, *n* (%)	No	371 (74)	91 (94)	134 (96)	73 (74)	46 (55)	27 (33)	< 0.001^*^
	Yes	129 (26)	6 (6)	5 (4)	26 (26)	38 (45)	54 (67)	
Bronchial cut-off sign, *n* (%)	No	461 (92)	97 (100)	139 (100)	96 (97)	77 (92)	52 (64)	< 0.001^*^
	Yes	39 (8)	0 (0)	0 (0)	3 (3)	7 (8)	29 (36)	
Speculation sign, *n* (%)	No	373 (75)	91 (94)	128 (92)	76 (77)	42 (50)	36 (44)	< 0.001^*^
	Yes	127 (25)	6 (6)	11 (8)	23 (23)	42 (50)	45 (56)	
Vacuole sign, *n* (%)	No	389 (78)	85 (88)	122 (88)	73 (74)	61 (73)	48 (59)	< 0.001^*^
	Yes	111 (22)	12 (12)	17 (12)	26 (26)	23 (27)	33 (41)	
Air bronchogram sign, *n* (%)	No	397 (79)	87 (90)	126 (91)	77 (78)	56 (67)	51 (63)	< 0.001^*^
	Yes	103 (21)	10 (10)	13 (9)	22 (22)	28 (33)	30 (37)	
Vascular convergence sign, *n* (%)	No	413 (83)	97 (100)	128 (92)	83 (84)	62 (74)	43 (53)	< 0.001^*^
	Yes	87 (17)	0 (0)	11 (8)	16 (16)	22 (26)	38 (47)	
Pleural retraction, *n* (%)	No	264 (53)	86 (89)	99 (71)	39 (39)	29 (35)	11 (14)	< 0.001^*^
	Yes	236 (47)	11 (11)	40 (29)	60 (61)	55 (65)	70 (86)	
Abnormal vessels within nodules, *n* (%)	No	326 (65)	69 (71)	110 (79)	45 (45)	51 (61)	51 (63)	< 0.001^*^
	Yes	174 (35)	28 (29)	29 (21)	54 (55)	33 (39)	30 (37)	
Nodule type, *n* (%)	pGGN	166 (33)	69 (71)	74 (53)	19 (19)	4 (5)	0 (0)	< 0.001^*^
	mGGN	239 (48)	25 (26)	63 (45)	73 (74)	51 (61)	27 (33)	
	SN	95 (19)	3 (3)	2 (1)	7 (7)	29 (35)	54 (67)	

*AIS* adenocarcinoma in situ, *MIA* minimally invasive adenocarcinoma, *WIAC* well-differentiated invasive adenocarcinoma, *MIAC* moderately differentiated invasive adenocarcinoma, *PIAC* poorly differentiated invasive adenocarcinoma, *pGGN* pure ground-glass nodule, *mGGN* mixed ground-glass nodule, *SN* solid nodules, *RUL* right upper lobe, *RML* right middle lobe, *RLL* right lower lobe, *LUL* left upper lobe, *LLL* left lower lobe

*p value was calculated with the Mann–Whitney *U* test < 0.05

^§^*p* value was calculated with the Chi-square test < 0.05

Consequently, two-classification system comprised non-IAC (AIS, MIA, *n* = 236) and IAC (WIAC, MIAC, and PIAC, *n* = 264). The three-classification system comprised non-IAC (AIS, MIA, *n* = 236), low-grade IAC (WIAC, MIAC, *n* = 183), and high-grade IAC (PIAC, *n* = 81). In the five-classification system, the subtypes included AIS (*n* = 97), MIA (*n* = 139), WIAC (*n* = 99), MIAC (*n* = 84), and PIAC (*n* = 81).

### The selected radiomics and clinic-radiological features in varying classification models

After extracting a total of 1688 radiomics features, only those demonstrating good feature consistency (ICC≧0.75) were selected for further analysis. For the two-classification model, a combination of variance thresholding, SelectKBest, and LASSO regression methods was utilized to identify 31 non-zero coefficient radiomics features from the CT sequences. These selected features were then used to calculate the 2-Rad-score for each patient in both the training and testing cohorts. The calculation of the 2-Rad-score involved summing the products of the corresponding feature values and their respective weights. Similarly, for the 17 clinic-radiological features, LASSO regression was employed to extract 11 non-zero coefficient clinic-radiological features. This process resulted in the computation of the 2-clinic-radiological-score using the same methodology.

Following the same approach, the 3-rad-score and 3-clinic-radiological-score for the three-classification model were calculated using 39 retained radiomics features and 14 retained clinic-radiological features, respectively. Additionally, for the five-classification model, 26 radiomics features and 11 clinic-radiological features were preserved to calculate the 5-rad-score and 5-clinic-radiological-score, respectively.

### Performance of the ML models in different classifications

Clinic-radiological, radiomics, and combined models were constructed for the two-classification task using LR, SVM, KNN, DT, RF, and GBDT algorithms. The performance of these ML models in the testing cohort was evaluated, and the results are presented in Table 3. All ML models exhibited satisfactory performance in predicting non-IAC and IAC. The SVM model showed the best overall performance, achieving the highest AUC and ACC values. Notably, the combined model demonstrated improved performance compared to the clinic-radiological and radiomics models, achieving an AUC of 0.942 and an ACC of 0.894, whereas the clinic-radiological and radiomics models achieved AUC values of 0.905 and 0.938, and ACC values of 0.848 and 0.868, respectively.

**Table 3:** The performance of ML models in testing cohort of two-classification model

Cohort	ML model	Clinic-radiological model				Radiomics model				Combined model			
		AUC	Accuracy	Sensitivity	Specificity	AUC	Accuracy	Sensitivity	Specificity	AUC	Accuracy	Sensitivity	Specificity
Train	LR	0.907	0.828	0.821	0.836	0.975	0.914	0.891	0.939	0.972	0.891	0.875	0.909
	SVM	0.932	0.874	0.864	0.885	0.962	0.877	0.848	0.909	0.985	0.934	0.924	0.945
	KNN	0.918	0.848	0.815	0.885	0.967	0.900	0.902	0.897	0.972	0.903	0.864	0.945
	DT	0.900	0.837	0.793	0.885	0.943	0.903	0.880	0.927	0.966	0.937	0.967	0.903
	RF	0.891	0.819	0.799	0.842	0.968	0.900	0.886	0.915	0.961	0.891	0.891	0.891
	GBDT	0.913	0.847	0.900	0.788	0.955	0.874	0.821	0.933	0.960	0.905	0.880	0.933
Test	LR	0.905	0.815	0.813	0.817	0.937	0.854	0.800	0.915	0.934	0.868	0.863	0.873
	SVM	0.905	0.848	0.838	0.859	0.938	0.868	0.825	0.915	**0.942**	**0.894**	**0.875**	**0.915**
	KNN	0.843	0.781	0.725	0.845	0.925	0.841	0.838	0.845	0.933	0.881	0.863	0.901
	DT	0.868	0.788	0.688	0.901	0.848	0.834	0.825	0.845	0.815	0.781	0.775	0.789
	RF	0.909	0.828	0.800	0.859	0.927	0.848	0.863	0.831	0.940	0.881	0.900	0.859
	GBDT	0.905	0.836	0.858	0.812	0.932	0.861	0.788	0.944	0.941	0.867	0.837	0.901

Bold values indicated the best performance in the two-classification task

*ML* machine learning, *LR* logistic regression, *SVM* support vector machine, *KNN* K-nearest neighbor, *DT* decision tree, *RF* random forest, *GBDT* gradient boosting decision tree, *AUC* area under the curve

Similarly, for the three-classification task, clinic-radiological, radiomics, and combined models were constructed using LR-OVR, SVM-OVR, LR-OVO, SVM-OVO, KNN, DT, RF, and GBDT algorithms. The performance of these models in predicting non-IAC, low-grade IAC, and high-grade IAC was evaluated, and the results are presented in Table 4. The SVM-OVO model demonstrated the best overall performance among all ML models in the testing cohort, regardless of the clinic-radiological, radiomics, or combined model. However, the combined model achieved a higher ACC compared to the other two models, with a value of 0.767 versus 0.740 and 0.753, respectively.

**Table 4:** The performance of ML models in testing cohort of three-classification model

Cohort	ML model	Clinic-radiological model				Radiomics model				Combined model			
		Accuracy	F1-score	Recall	Precision	Accuracy	F1-score	Recall	Precision	Accuracy	F1-score	Recall	Precision
Train	LR-OVR	0.723	0.704	0.737	0.696	0.809	0.781	0.789	0.777	0.837	0.823	0.834	0.816
	SVM-OVR	0.451	0.389	0.461	0.410	0.794	0.773	0.790	0.765	0.723	0.699	0.697	0.702
	LR-OVO	0.737	0.711	0.705	0.721	0.849	0.836	0.825	0.851	0.871	0.869	0.865	0.873
	SVM-OVO	0.749	0.721	0.713	0.734	0.846	0.830	0.820	0.842	0.883	0.878	0.870	0.886
	DT	0.723	0.700	0.704	0.696	0.820	0.743	0.726	0.876	0.820	0.743	0.726	0.876
	KNN	0.734	0.705	0.680	0.777	0.797	0.750	0.733	0.784	0.777	0.741	0.724	0.771
	RF	0.749	0.717	0.695	0.770	0.851	0.822	0.806	0.849	0.851	0.825	0.804	0.866
	GBDT	0.789	0.734	0.714	0.789	0.780	0.721	0.701	0.788	0.763	0.745	0.738	0.753
Test	LR-OVR	0.713	0.672	0.710	0.673	0.740	0.702	0.703	0.707	0.733	0.696	0.697	0.701
	SVM-OVR	0.413	0.333	0.388	0.361	0.713	0.674	0.688	0.670	0.693	0.633	0.630	0.641
	LR-OVO	0.733	0.695	0.687	0.710	0.747	0.708	0.709	0.711	0.747	0.702	0.693	0.715
	SVM-OVO	0.740	0.696	0.697	0.700	0.753	0.712	0.706	0.724	**0.767**	**0.718**	**0.710**	**0.731**
	DT	0.707	0.680	0.682	0.677	0.653	0.563	0.555	0.669	0.653	0.562	0.555	0.643
	KNN	0.627	0.561	0.545	0.640	0.633	0.572	0.577	0.577	0.673	0.598	0.589	0.634
	RF	0.713	0.660	0.646	0.696	0.707	0.639	0.629	0.672	0.700	0.636	0.629	0.652
	GBDT	0.700	0.635	0.625	0.667	0.713	0.645	0.635	0.678	0.720	0.672	0.652	0.727

Bold values indicated the best performance in the three-classification task

*ML* machine learning, *OVR* one versus rest, *OVO* one versus one, *LR* logistic regression, *SVM* support vector machine, *KNN* K-nearest neighbor, *DT* decision tree, *RF* random forest, *GBDT* gradient boosting decision tree

The results of the five-classification task, as presented in Table 5, were consistent with the three-classification results. The SVM-OVO model exhibited the best overall performance in predicting AIS, MIA, WIAC, MIAC, and PIAC. Once again, the combined model outperformed the other two models in terms of ACC, achieving a value of 0.607 compared to 0.513 and 0.553.

**Table 5:** The performance of ML models in testing cohort of five-classification model

Cohort	ML model	Clinic-radiological model				Radiomics model				Combined model			
		Accuracy	F1-score	Recall	Precision	Accuracy	F1-score	Recall	Precision	Accuracy	F1-score	Recall	Precision
Train	LR-OVR	0.500	0.484	0.491	0.485	0.597	0.592	0.595	0.591	0.631	0.623	0.626	0.623
	SVM-OVR	0.271	0.184	0.267	0.170	0.666	0.667	0.670	0.676	0.426	0.426	0.446	0.438
	LR-OVO	0.509	0.486	0.488	0.501	0.626	0.618	0.618	0.624	0.683	0.670	0.670	0.676
	SVM-OVO	0.557	0.539	0.534	0.613	0.700	0.696	0.685	0.722	0.703	0.693	0.690	0.715
	DT	0.523	0.488	0.491	0.605	0.651	0.624	0.635	0.714	0.663	0.644	0.649	0.696
	KNN	0.574	0.566	0.554	0.625	0.637	0.627	0.622	0.645	0.609	0.612	0.600	0.635
	RF	0.537	0.503	0.512	0.531	0.620	0.613	0.611	0.620	0.689	0.683	0.674	0.705
	GBDT	0.523	0.462	0.491	0.545	0.694	0.685	0.677	0.732	0.700	0.692	0.686	0.722
Test	LR-OVR	0.420	0.416	0.427	0.415	0.467	0.440	0.475	0.429	0.520	0.507	0.531	0.500
	SVM-OVR	0.287	0.191	0.283	0.166	0.500	0.478	0.502	0.474	0.427	0.416	0.445	0.421
	LR-OVO	0.453	0.446	0.452	0.448	0.493	0.462	0.483	0.453	0.547	0.528	0.550	0.522
	SVM-OVO	0.513	0.506	0.505	0.514	0.553	0.531	0.536	0.559	**0.607**	**0.594**	**0.603**	**0.593**
	DT	0.500	0.455	0.467	0.539	0.480	0.448	0.471	0.460	0.500	0.490	0.494	0.502
	KNN	0.427	0.417	0.407	0.462	0.453	0.438	0.444	0.446	0.460	0.441	0.443	0.442
	RF	0.467	0.429	0.454	0.423	0.507	0.497	0.500	0.505	0.527	0.499	0.507	0.513
	GBDT	0.440	0.378	0.419	0.412	0.493	0.457	0.479	0.480	0.500	0.460	0.484	0.478

Bold values indicated the best performance in the five-classification task

*ML* machine learning, *OVR* one versus rest, *OVO* one versus one, *LR* logistic regression, *SVM* support vector machine, *KNN* K-nearest neighbor, *DT* decision tree, *RF* random forest, *GBDT* gradient boosting decision tree

### Optimal ML model classification evaluation

**Fig. 3:**
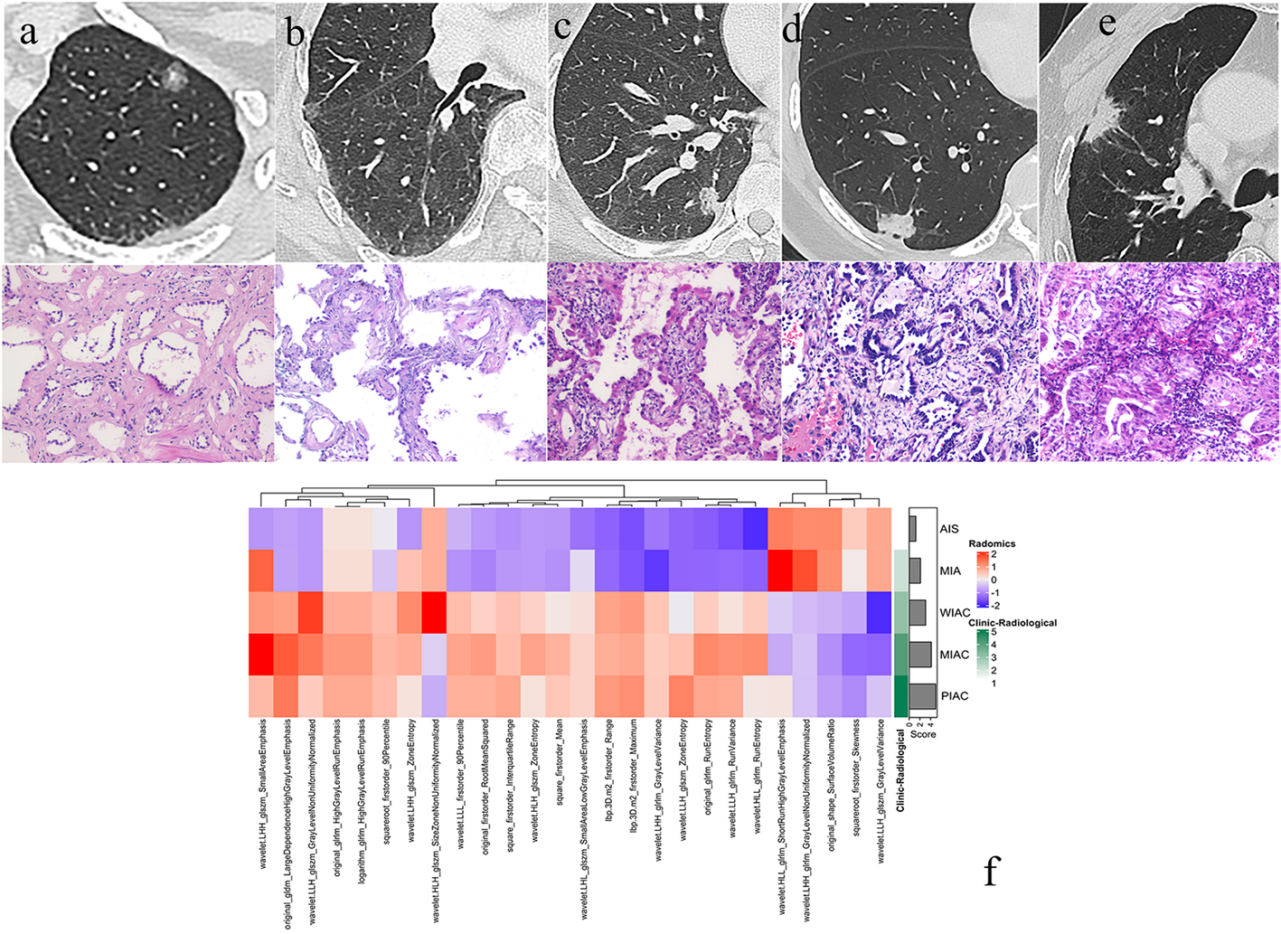
The cluster graph provided a visual representation of the relative correlation between radiomics features, clinic-radiological scores, and histological types in a set of representative patients. CT images (**a**–**e** upper) and corresponding histological images (**a**–**e** lower, magnification × 200) of five representative patients are displayed. Patient 1, a 53-year-old woman with adenocarcinoma in situ (AIS), exhibited a CT image in the left upper lobe showing a ground-glass nodule (GGN) within a foci solid component (**a**). Patient 2, a 74-year-old woman with minimally invasive adenocarcinoma (MIA), presented a CT image in the right upper lobe displaying a GGN within a blurred vessel (**b**). Patient 3, a 58-year-old woman with well-differentiated invasive adenocarcinoma (WIAC), demonstrated a CT image in the right lower lobe depicting a mixed GGN with lobulation, short speculation, and pleural retraction (**c**). Patient 4, a 53-year-old woman with moderately differentiated invasive adenocarcinoma (MIAC), showed a CT image in the right lower lobe exhibiting a solid nodule (SN) with lobulation, short speculation, and pleural retraction (**d**). Patient 5, a 70-year-old man with poorly differentiated invasive adenocarcinoma (PIAC), presented a CT image in the right upper lobe revealing an SN with lobulation, more short speculation, pleural retraction, and vascular convergence (**e**). Furthermore, a comparison of 36 radiomics features and the clinic-radiological score among the five patients is illustrated in panel (**f**)

The SVM-OVO ML model, combined with radiomics and clinic-radiological features, exhibited excellent performance in classifying pulmonary adenocarcinoma nodules, as presented in Table 6. To visualize the correlation between radiomics features, clinic-radiological scores, and histological types, a cluster graph was constructed using representative patient data, as shown in Fig. 3.

**Table 6:** The performance of multi-classification using SVM-OVO for predicting histological classification of pulmonary adenocarcinoma nodules

Cohort	Model	Two-classification				Three-classification				Five-classification			
		AUC	Accuracy	Sensitivity	Specificity	Accuracy	F1-score	Recall	Precision	Accuracy	F1-score	Recall	Precision
Train	Clinic-radiological	0.932	0.874	0.864	0.885	0.749	0.721	0.713	0.734	0.557	0.539	0.534	0.613
	Radiomics	0.962	0.877	0.848	0.909	0.846	0.830	0.820	0.842	0.700	0.696	0.685	0.722
	Combined	0.985	0.934	0.924	0.945	0.883	0.878	0.870	0.886	0.703	0.693	0.690	0.715
Test	Clinic-radiological	0.905	0.848	0.838	0.859	0.740	0.696	0.697	0.700	0.513	0.506	0.505	0.514
	Radiomics	0.938	0.868	0.825	0.915	0.753	0.712	0.706	0.724	0.553	0.531	0.536	0.559
	Combined	**0.942**	**0.894**	**0.875**	**0.915**	**0.767**	**0.718**	**0.710**	**0.731**	**0.607**	**0.594**	**0.603**	**0.593**

Bold values indicated the best performance in the multi-classification task

*AUC* area under the curve

**Fig. 4:**
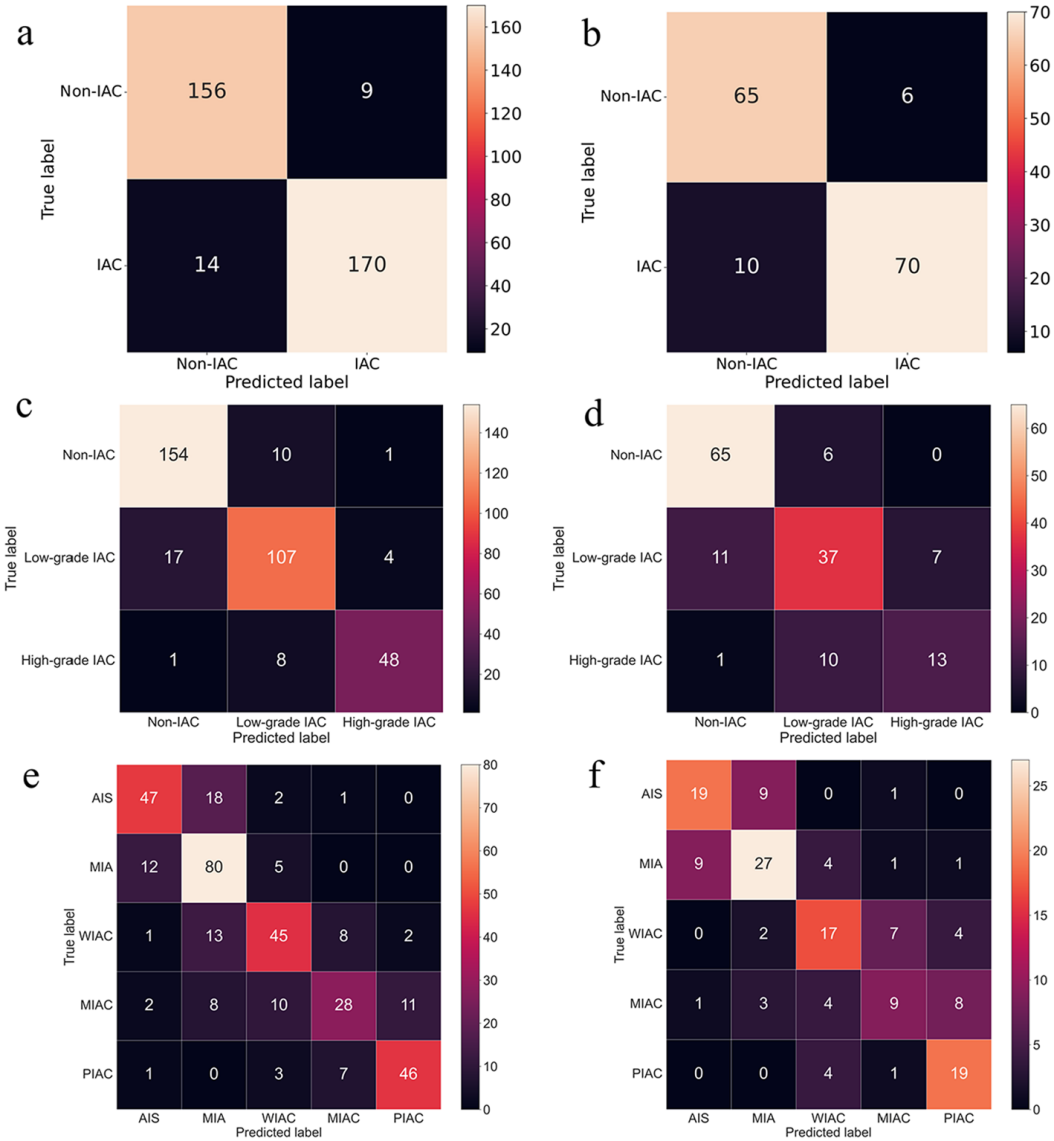
Confusion matrix on radiomics combined model: the two-classification in train cohort (**a**) and test cohort (**b**), the three-classification in train cohort (**c**) and test cohort (**d**), and the five-classification in train cohort (**e**) and test cohort (**f**)

**Fig. 5:**
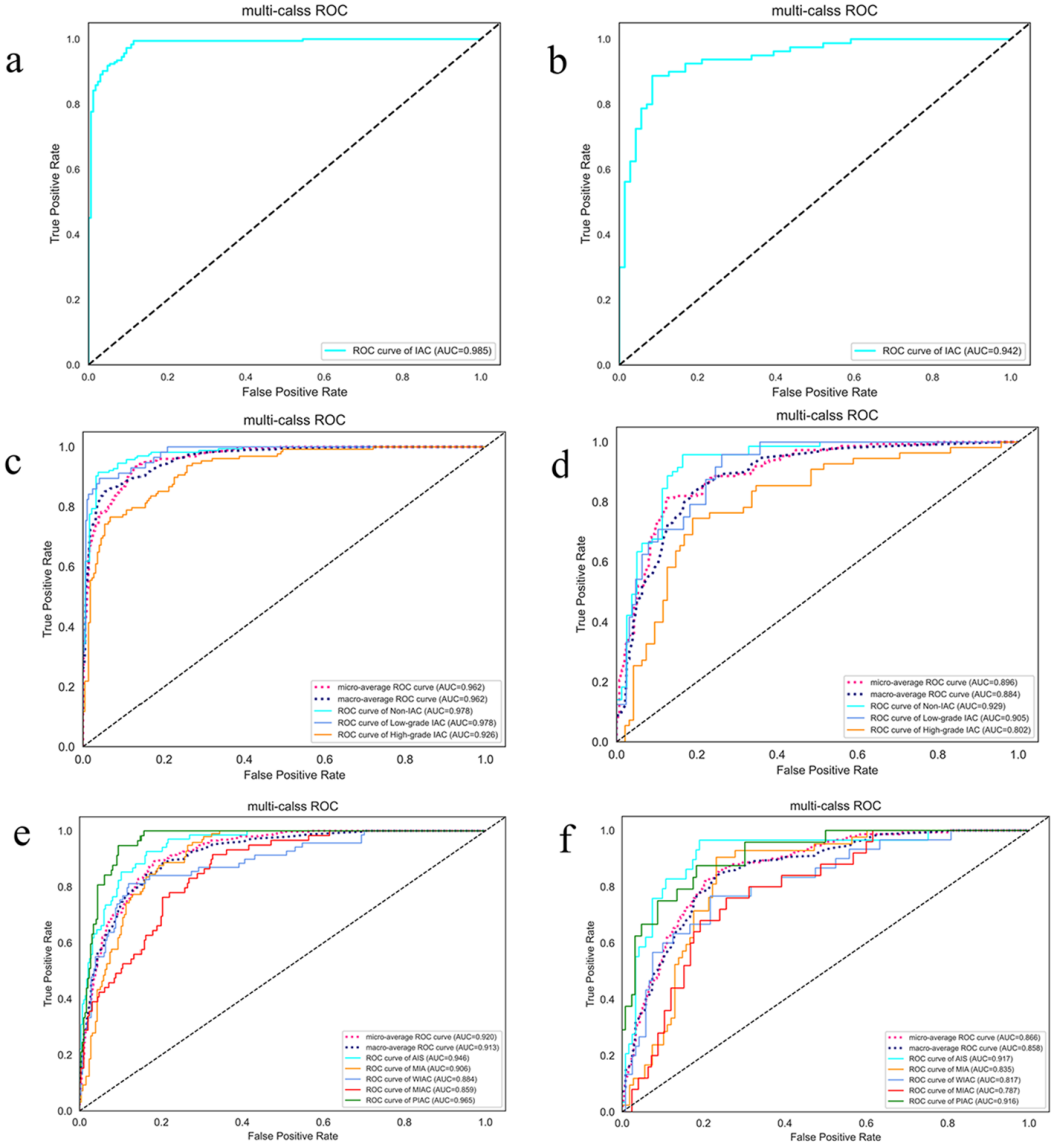
ROC curve on radiomics combined model: the two-classification in train cohort (**a**) and test cohort (**b**), the three-classification in train cohort (**c**) and test cohort (**d**), and the five-classification in train cohort (**e**) and test cohort (**f**)

Figure 4 displays the confusion matrix of the combined model using SVM and SVM-OVO in both the training and testing cohorts. The matrix illustrates that the selected models were not prone to making errors and effectively captured the relationships among histological subtypes. The ACC in the testing cohort exceeded 0.6, even for the challenging five-classification task. All histological subtypes in the three and five-classification tasks were accurately identified. The macro-AUC and micro-AUC values of the three-classification model in the testing cohort were 0.884 and 0.896, respectively. Similarly, the macro-AUC and micro-AUC values of the five-classification model were 0.858 and 0.866, respectively. The AUC values of the histological subtypes ranged from 0.787 to 0.942, with the lowest AUC observed for MIAC in the testing cohort, as depicted in Fig. 5.

## Discussion

In this research endeavor, our objective was to devise classification models for two, three, and five histological stratifications of pulmonary adenocarcinoma nodules by integrating radiomics features with clinic-radiological characteristics. Additionally, we conducted a comprehensive comparison of various machine learning techniques, including LR-OVR, SVM-OVR, LR-OVO, SVM-OVO, DT, KNN, RF, and GBDT, to identify the most suitable model for multi-classification tasks in predicting the histological subtypes of pulmonary nodules. Consequently, the SVM-OVO model emerged as the optimal choice, exhibiting superior overall performance in accurately predicting the histological subtypes of pulmonary nodules.

Undoubtedly, the accurate histological classification of LAC, which evaluates invasiveness and differentiation, is pivotal in determining appropriate treatment strategies. Typically, this classification relies on postoperative pathological examination following complete surgical resection [9, 37]. Consequently, the development of a non-invasive and convenient method to predict the histological classification of pulmonary nodules based on preoperative CT images holds substantial clinical significance. Radiomics, as a burgeoning field, has shown great promise in the diagnosis, treatment, and monitoring of pulmonary nodules, surpassing the capabilities of radiologist-based assessments, as evidenced by a growing body of literature [15, 21, 22, 38].

In our present investigation, we put forth multiple common machine learning models encompassing the diverse histopathologic stratifications of pulmonary adenocarcinoma nodules, as per the fifth WHO classification of lung tumors. This encompassed the subtypes of AIS, MIA, WIAC, MIAC, and PIAC. Upon evaluating their discriminatory abilities, we observed that the SVM model yielded the most favorable outcomes in the testing cohort for the two-classification task, while SVM-OVO demonstrated superior performance among the ML models in the testing cohort for the three- and five-classification tasks. The area under the AUC ranged from 0.787 to 0.942, and the ACC ranged from 0.607 to 0.894. Notably, our findings align with previous research that highlighted the satisfactory performance of classifying pulmonary adenocarcinoma nodules in the two-classification task [22, 25, 39]. Furthermore, our results indicate that the ACC achieved in the five-classification task surpassed the previous multi-classification of the predominant histologic pattern [40].

However, previous studies focusing on the classification of pulmonary nodules have generally overlooked the crucial aspect of selecting appropriate machine learning models. In light of this gap, our study sought to compare various models and assess their performance in classifying pulmonary nodules. Encouragingly, our findings aligned with prior research, demonstrating that the SVM outperformed other machine learning models in the testing cohort. SVM, widely employed for classification and predictive modeling tasks, has established itself as a reliable choice, even when confronted with limited data availability [41–43].

In the realm of multi-class classification, two widely adopted strategies are the OVO and OVR approaches. Park et al. utilized the OVO method to predict the three primary subtypes of lung adenocarcinoma, as described in their study [21]. Similarly, Chen et al. employed the OVR method to construct a three-classification model for the preoperative prediction of risk stratification in gastrointestinal stromal tumors [36]. In another investigation by Liu et al., a four-classification OVO model was established to differentiate subtypes of non-small cell lung cancer [44]. When comparing the performance of the ML models for multi-classification, it was consistently observed that the OVO approach outperformed the OVR method in the testing cohort, which aligns with the findings reported by Liu et al.

Previous studies have also highlighted the correlation between clinic-radiological features and the histological classification of pulmonary nodules. Consequently, experienced radiologists can utilize clinic-radiological features to classify the pathologic subtypes of pulmonary nodules [2, 21, 45]. Certain unique or characteristic features, such as the presence of minute airspaces or dilated vessels within the lesions, play a significant role in nodule classification. However, these specific features may not exhibit a correlation with the radiomics feature category [46]. To enhance the predictive capability of the radiomics model for the classification of pulmonary nodules, we developed a combined model that incorporates both radiomics and clinic-radiological features. Our study demonstrated that the predictive power of the combined model surpassed that of the radiomics and clinic-radiological models, regardless of whether the classification task involved two classes or multiple classes.

The predictive performance of high-grade IAC in the testing cohort of three-classification combined models was found to be lower compared to non-IAC and low-grade IAC, which is in contrast to the results obtained from the five-classification model. This discrepancy may be attributed to the relatively lower number of high-grade IAC patients, resulting in data bias. Additionally, the predictive power of MIAC in the five-classification combined model was lower than that of other subtypes. This could be attributed to the insufficient sample size, leading to uncertainties in the analysis and slight variations in the proportion of invasive components within some MIAC patients.

It is important to acknowledge several limitations of the present study. First, the study's retrospective design, utilization of a single central dataset, and absence of external validation may limit the generalizability of the developed models. Second, the relatively small sample size may impact the statistical power of the analysis. Third, the incorporation of deep learning and improvement of machine learning models are necessary as more data become available. Fourth, the presence of spread through air spaces, which is commonly observed in patients with invasive adenocarcinoma, is closely linked to patient prognosis. Therefore, further investigations are warranted to establish additional radiomics classifications for predicting pathological characteristics.

In conclusion, this study underscores the importance of selecting appropriate machine learning models and demonstrates the utility of multi-classification radiomics combined with clinic-radiological features in predicting the invasiveness and differentiation of pulmonary adenocarcinoma nodules. The SVM-OVO model for the multi-classification task exhibited the best overall performance and successfully predicted the histological stratification of non-invasive subtypes.

## Data Availability

The datasets generated during and/or analyzed during the current study are available from the corresponding author on reasonable request.

## Abbreviations

AIS: Adenocarcinoma in situ
MIA: Minimally invasive adenocarcinoma
IAC: Invasive adenocarcinoma
WIAC: Well-differentiated invasive adenocarcinoma
MIAC: Moderately differentiated invasive adenocarcinoma
PIAC: Poorly differentiated invasive adenocarcinoma
LASSO: Least absolute shrinkage and selection operator
ML: Machine learning
ACC: Accuracy
AUC: Area under the curve
SVM: Support vector machine
SVM-OVO: One versus one strategy of SVM
LAC: Lung adenocarcinoma
GGN: Ground glass nodule
SN: Solid nodule
ROI: Region of interest
GLCM: Gray-level co-occurrence matrix
GLRL: Gray-level run length matrix
GLSZM: Gray-level size zone matrix
NGTDM: Neighboring gray tone difference matrix
GLDM: Gray-level dependence matrix
LR: Logistic regression
KNN: K-nearest neighbors
DT: Decision tree
RF: Random forest
GBDT: Gradient boosting decision tree
SVM-OVR: One versus rest strategy of SVM
ICC: Intraclass correlation coefficient
